# Impact of high-speed rail opening on the site selection of renewable energy enterprises: empirical evidence from China

**DOI:** 10.1186/s13021-025-00321-4

**Published:** 2025-08-26

**Authors:** Wenjun Han, Xianhua Wu, Yiting Wang, Shiyun Huang

**Affiliations:** 1 School of Economics and Management, Shanghai Maritime University, Shanghai, 201306 China; 2 Shanghai Zhenbei Middle School, Shanghai, 201306 China

**Keywords:** Difference-in-differences, High-speed rail, Renewable energy enterprises, Site selection

## Abstract

High-speed rail is a crucial transportation infrastructure in China and has a significant impact on the location choice of renewable energy enterprises. However, it is unclear how substantial is this impact, and how can it be measured. Research on these topics is quite limited. This study collects the data on various prefecture-level cities in China and their renewable energy enterprises from 2006 to 2020. Using high-speed rail openings as a dummy variable and a multi-period Difference-in-Differences (DID) approach, this study empirically examines the effect of high-speed rail openings on the site selection of renewable energy enterprises. The findings indicate that: (1) The coefficient for the impact of high-speed rail openings on the site selection of renewable energy enterprises is significantly positive, suggesting that high-speed rail policies facilitate cites’ acceptance of these enterprises; (2) There is a regional imbalance in the site selection for renewable energy enterprises, with economically developed cities showing greater receptivity; (3) Cities with higher waste treatment rates tend to attract more renewable energy enterprises.

## Introduction

With the development of the economy, countries around the world have been actively constructing high-speed railways. In 1964, Japan launched the Tōkaidō Shinkansen, becoming the first country in the world to operate high-speed rail. Subsequently, Japan built a total of 1850 km of rail lines.^1^ In 1981, France’s Southeast High-Speed Railway (train à grande vitesse, TGV) began operations. In 1990, the TGV set a world record by reaching a speed of 515.3 km/h.^2^ In 1991, Germany opened its first domestic high-speed railway, the InterCity Express (ICE), with a maximum speed of 320 km/h. Today, the ICE serves all major cities within Germany and has international connections with countries.^3^ In 1998, the Guangzhou-Shenzhen Railway became China’s first high-speed rail line to meet operational speed standards, achieving a maximum speed of 200 km/h. In 2002, the maglev railway line was completed, with a speed of up to 430 km/h, marking China’s first high-speed rail system. Over the past two decades, the construction of high-speed rail lines in China has expanded significantly. In 2015, China and Russia initiated a railway construction project for the Moscow-Kazan section. In 2017, China partnered with Indonesia on the Jakarta-Bandung railway project. From 2001 to 2019, the maximum operational speed of China’s high-speed rail increased from 200 km/h to 350 km/h, making it the fastest in the world.^4^ A report published by the World Bank in 2019 titled “The Development of High-Speed Rail in China” noted that the operational mileage of China’s high-speed rail exceeds the combined total of all other countries’ high-speed rail systems.

The rapid development of high-speed rail (HSR) in China has spurred investment and employment in related industries and boosted economic growth in regions with underdeveloped transportation systems. Lu Wei, researcher at the Land Institute of the Chinese Academy of Macroeconomic Research, states that a one-billion-CNY investment in HSR construction stimulates directly three billion CNY of investment in associated industries, including metallurgy and manufacturing. This investment generates approximately 2200 jobs. From the perspective of regional economic development, HSR can foster economic growth in areas with limited transportation options. For example, the launch of HSR lines has facilitated the growth of the logistics industry in Sichuan, Gansu, and Shaanxi provinces, with 55 e-commerce and express delivery companies joining a union on the very day of the rail’s inauguration. The opening of the Bao-Lan High-Speed Railway connected cities along the ancient Silk Road, generating around 70 million CNY in tourism revenue within the first year of operation.^5^ The above data indicate that the development of high-speed rail can promote regional economic growth and, in turn, encourage enterprises to establish local facilities. Therefore, it is crucial to analyze the impact of high-speed rail on enterprise location decisions. This study focuses on new energy enterprises as the research subject to investigate how the opening of HSR affects their location choices and agglomeration patterns. This leads to the questions: What specific effects does the HSR lines have on the distribution of new energy enterprises? How substantial are these effects? Additionally, what measures should the government undertake to support the development of new energy enterprises in this context? To address these questions, it is essential to investigate how HSR lines influence the location choices of new energy enterprises. This research holds significant practical value for understanding the investment behaviors and dynamic layouts of these enterprises and promoting their better and faster development.

The swift expansion of HSR has garnered significant attention from scholars. As an emerging transportation mode in the logistics sector, HSR provides abundant transportation capacity resources and can accommodate logistics demands characterized by scale, high added value, and personalization [32]. When the Red Sea Waterway is blocked, HSR can replace sea transportation and complete the cargo transportation work between China and Europe  [26]. The high-speed rail express system is recognized as one of the most promising logistics systems [31]. The launch of high-speed railways facilitates the development of the express delivery industry [12] and addresses the growing demand for express cargo services [30]. However, while promoting industrial agglomeration and enhancing green technological innovation, the operation of high-speed railways has exacerbated urban environmental pollution in China [6]. Research shows that the low-carbon efficiency of China's overall railway-waterway intermodal transportation needs further improvement [35].

Research on transportation infrastructure primarily concentrates on two dimensions. Certain researches have examined the construction of transportation infrastructure and its impacts on energy, society, and the economy. The environmental effects of transportation infrastructure exhibit heterogeneity. From a carbon reduction perspective, such infrastructure exerts the most pronounced emission-reduction effects in western China, followed by the central region. In eastern China, however, transportation infrastructure demonstrates insignificant support for carbon mitigation [22, 23]. When economic agglomeration levels are low, transportation infrastructure exerts no discernible impact on changes in green total factor productivity. As agglomeration intensifies, however, this impact becomes statistically significant [19]. Optimization of transportation infrastructure reveals an energy-saving potential of 12.22–29.35 million kilowatt-hours [18]. Nevertheless, expanded transportation infrastructure (e.g., buses and subways) in certain regions may adversely affect urban air quality [9]. Studies in Southeast Asia indicate that increased pressure on transport infrastructure within forested World Heritage Sites may precipitate forest degradation and biodiversity loss [16]. From socioeconomic perspectives, transportation infrastructure positively influences urbanization, metropolitan statistical area employment, and urban productivity enhancement [14, 17, 21]. Strategic adjustment of transportation infrastructure investment can alleviate traffic congestion, thereby affecting social welfare [1]. Furthermore, enhanced transportation infrastructure amplifies the stimulative effect of digital payments on tourism consumption expenditure [3].

Additionally, some researches have focused on how changes in external environments and emerging technologies affect transportation infrastructure. The implementation of advanced sensors and novel artificial intelligence methodologies has enhanced the service convenience of public transportation systems while extending the operational lifespan of transportation infrastructure [8]. However, the adoption of emerging technologies such as digital twin technology may also present challenges to transportation infrastructure integrity [2]. From an environmental change perspective, under a projected end-of-21st-century temperature increase of 4 °C, 43.6% of global transportation infrastructure is anticipated to experience at least a 69.9% reduction in functionality during extreme rainfall events [13].

Research on enterprise location selection predominantly examines the following dimensions. National policies significantly influence corporate location decisions. The impact of central bank independence on location choices is more pronounced for non-state-owned enterprises than for state-owned counterparts [29]. Government information disclosure demonstrates greater attractiveness to environmentally benign enterprises while exerting inhibitory effects on polluting firms [15]. Enhanced low-carbon regulatory intensity in urban areas correlates positively with entry willingness among high-productivity enterprises [33]. Enterprises exhibit stronger investment preferences toward regions with industrial policy support [5]. Increased power resource allocation directly facilitates enterprise location transfers [25]. Traditional determinants including transportation and market conditions substantially affect location selection. Subway network development positively influences service enterprise location choices, with new subway openings correlating with a 53.8% increase in registered service enterprises [7]. Regions exhibiting superior demand accessibility demonstrate significantly heightened attractiveness to digital enterprises [24]. Beyond traditional determinants, emerging location factors exert increasingly significant impacts on new digital enterprises' location selection [27]. The influence of emerging factors-such as informatization levels and financial development-on port and shipping service enterprises’ location choices is growing in complexity [11]. From transnational perspectives, foreign investment positively affects enterprise site selection in emerging markets [10]. However, elevated political risks deter Chinese small and medium-sized enterprises from establishing operations in host countries [4].

A review of extant literature reveals that most scholars have concentrated on examining how HSR construction facilitates development in the express delivery, logistics, and transportation sectors. However, empirical quantitative analyses addressing the impact of HSR development on industrial location decisions remain limited. Furthermore, academic investigation into the influence of transportation infrastructure advancement on new energy enterprise site selection has received less attention. Additionally, research exploring the effects of HSR operations on corporate location choices has been understudied. Amid growing societal emphasis on environmental protection accompanying economic development, academic inquiry into the relationship between emerging new energy industries and transportation infrastructure evolution-particularly HSR-remains insufficient. This study aims to address these research gaps by analyzing how HSR inaugurations affect location selection patterns among new energy enterprises, thereby enriching the corpus of knowledge on transportation infrastructure impacts.

The remaining sections of this paper are structured as follows: “Econometric model and data description” section presents three hypotheses based on theoretical analysis and introduces a difference-in-differences model to analyze the relevant data; “Benchmark regression and data analysis” section conducts baseline regression according to the model and variables, and verifies the robustness of the results through methods such as parallel trends, robustness checks, and heterogeneity analyses; “Conclusion and suggestions” section concludes the study and provides relevant policy implications.

## Econometric model and data description

### Research hypotheses

Classical location theory employs a static, partial equilibrium analysis to explore the optimal location choice of individual firms based on the principles of perfect competition and price theory. Classical location theory primarily focuses on local and static conditions and does not consider government intervention in economic activity location choices. However, it highlights the significant influence of product market prices, supply–demand relationships, competition, and transportation costs on site selection. This provides a foundational framework for subsequent theoretical developments. Building on classical theory, neoclassical location theory upgrades the focus on the optimal location choice from individual firms to the region scale. It incorporates multiple factors such as production, distribution, transportation, ecology, and policy into its analysis, emphasizing the interconnections among multiple location points. For example, experiments conducted on the input of raw materials and output of products across various geographical locations reveal that profit differentials arise due to variations in transportation expenses.

In both classical and neoclassical location theories, the firm location decisions are influenced by industry structure, pricing, production technology, exogenous factors (such as input–output freight rates), policies, and public facilities. The ultimate objective is to maximize producer profits and consumer utility. With the upgrading of transportation infrastructure (e.g., the opening of HSR), the locational advantages among cities change, resulting in reduced transportation costs and altered resource endowments, thereby influencing industry clustering effects. The reduction in transportation time and the enhancement of accessibility brought about by HSR significantly facilitate inter-industry supply chains and lower transportation costs. For renewable energy enterprises, the inauguration of HSR significantly reduces operational expenditures through indirect mechanisms. Regarding labor mobility, HSR enhances geographical labor mobility, thereby mitigating unnecessary costs associated with coordinating geographically dispersed projects. In terms of business interaction efficiency, HSR compresses business travel duration, consequently lowering temporal expenditures. From financing and supply chain coordination perspectives, HSR expedites interregional mobility of supply chain technicians, accelerating response capabilities while diminishing enterprise operational overhead. Owing to data source limitations, our dataset does not differentiate between renewable energy enterprise types or product categories, instead analyzing the sector holistically. Nevertheless, the cost-reduction mechanism facilitated by HSR exhibits universality across these enterprises. Specifically, HSR compresses temporal requirements for interregional mobility of technicians and managers, optimizes equipment logistics, strengthens interregional collaborative capacities, enhances R&D efficiency, lowers equipment maintenance overhead, and accelerates supply response velocity.

New economic geography theory takes transportation costs into consideration, addressing the limitations of neoclassical location theory. Under conditions of increasing returns to scale and imperfect competition, this theory introduces the peripheral theory, which posits that economies of scale and transportation costs are mutually influential. In the initial stage, a city’s geographical advantages attract different manufacturers from various regions, leading to changes in their location choices. Once a certain level of industrial concentration is achieved in a region, integrated regional economies develop rapidly, granting them a competitive advantage through regional monopoly.

**Fig. 1:**
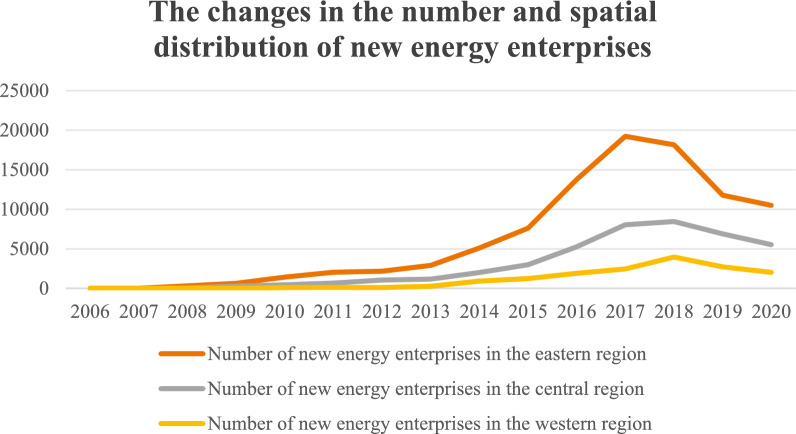
The changes in the number and spatial distribution of new energy enterprises

In recent years, China has witnessed a substantial increase in the number of renewable energy enterprises. As depicted in Fig. 1, the period from 2006 to 2020 reveals a peak in new energy enterprise registrations during 2017, followed by a decline from 2018 to 2020. Geographically, renewable energy enterprises demonstrate a pronounced regional concentration, with the highest density in eastern China and the lowest in western regions. The reason for this may be that the renewable energy industry relies on the development of related sectors. Therefore, the flow of products between upstream and downstream industries, as well as reduction of communication time between firms, is particularly important. A complete value chain and industrial chain for renewable energy can facilitate the sustainable development of renewable energy enterprises. The upstream sectors of renewable energy enterprises primarily encompass raw material extraction and R&D activities, which constitute foundational departments supplying essential inputs for production. Downstream industries exhibit considerable breadth across the sector. For instance, within the photovoltaic power generation industry, downstream segments include specialized power engineering sectors engaged in photovoltaic power station construction, operation, and maintenance. Similarly, for wind power generation, downstream industries involve energy corporations dedicated to wind farm development and operations. This configuration demonstrates the extensive value chain integration characteristic of renewable energy enterprise production processes. Therefore, transportation infrastructure, industrial agglomeration, and economic conditions are significant factors influencing corporate location selection. Similarly, renewable energy firms will consider maximizing profits and minimizing costs in their site selection. Therefore, the location choice for companies should comprehensively consider resource endowments, environmental regulations, transportation infrastructure, and industrial policies.

Based on the above analysis, the following hypotheses are proposed:

- Hypothesis 1: The opening of HSR increases the number of renewable energy enterprises in the host city.
- Hypothesis 2: The opening of HSR results in a greater number of new energy enterprises in economically developed cities compared with less developed ones.
- Hypothesis 3: In cities with higher levels of environmental awareness, the opening of HSR has a greater positive effect on the location selection of new energy enterprises in those regions.

### Econometric model

When estimating treatment effects, a situation may arise where there are multiple treated entities within the treatment group, and the timing of treatment implementation varies among them. Since the dates for the opening of HSR differ across cities, a multi-period difference-in-differences (DID) approach is appropriate. This study establishes a double DID model to analyze the impact of HSR openings on the site selection of renewable energy enterprises. The basic model is expressed as follows:

1 $$Y_{it} = \alpha + \beta HASRAILWAY_{it} + \varphi Control_{it} + \mu_{i} + \gamma_{t} + \varepsilon_{it}$$

In this model, i and t are city i and year t, respectively; ${\text{Y}}_{\text{it}}$ is the number of new renewable energy enterprises established in city i in year t; ${\text{HASRAILWAY}}_{\text{it}}$ is a dummy variable indicating the presence of HSR-assigned as 1 for cities where HSR is available and 0 otherwise. ${\text{Control}}_{\text{it}}$ variables include population, local economies of scale, industrial structure, foreign direct investment, financing potential, total freight transportation volume, and pollution levels. ${\upmu }_{\text{i}}$ and ${\upgamma }_{\text{t}}$ are individual fixed effects and time fixed effects, respectively. They can reflect time-specific and individual-specific characteristics and mitigate errors due to omitted variable bias. According to the multi-period DID model, β is the estimated coefficient that reflects the impact of HSR openings on the location choice of renewable energy enterprises.

### Data sources

Due to the exclusion of cities that either do not have HSR or renewable energy enterprises by the year 2020, this analysis selects a sample of 235 cities with both HSR and newly established renewable energy enterprises from 2006 to 2020. Data on renewable energy enterprises is sourced from the China Renewable Energy Enterprises Directory (https://industry.emagecompany.com/mine/xinnengyuan.html). Environmental data, including total population at year-end, per capita regional GDP, the proportion of the tertiary sector, employment figures, sulfur dioxide emissions, foreign direct investment amounts, and total freight volume, is obtained from the annual editions of the China Urban Statistical Yearbook and the China Statistical Yearbook.

### Variable selection

In economic activities, many factors influence the location choices of renewable energy enterprises. Based on neoclassical location theory and new economic geography, this study takes the number of newly established renewable energy enterprises in cities as the dependent variable, with the opening of HSR as the core independent variable. Control variables include population, local economies of scale, industrial structure, foreign direct investment, financing potential, total freight transportation volume, and pollution levels, which are employed to account for individual differences among cities.

1. Number of Newly Established Renewable Energy Enterprises: The quantity of newly established renewable energy enterprises in a city is regarded as the dependent variable. By monitoring the number of new renewable energy enterprises, the impact of various factors on the establishment of these enterprises can be assessed.
2. Opening of High-Speed Rail: The opening of HSR serves as the core independent variable in the model. A dummy variable is created based on whether HSR was available in 235 prefecture-level cities in China from 2006 to 2020, with the year of opening coded as “1” and the years without HSR coded as “0”. If a prefecture-level city has multiple HSR lines, the earliest opening year is recorded.
3. Population: The population size determines whether a city has an adequate labor supply. A larger population enhances the availability of labor in the market. In areas with a shortage of labor, labor prices tend to rise, which is detrimental to the development of new energy enterprises. When labor is insufficient, new energy enterprises may face difficulties in hiring, leading to operational disruptions. In contrast, a rich population and sufficient labor force can reduce costs, save expenses for new energy enterprises, increase profit margins, and ultimately enhance the level of industrial agglomeration in the region.
4. Local Economies of Scale: Economically backward regions cannot achieve sustainable industrial development or provide adequate space and guarantees for enterprise construction and development. In contrast, economically robust areas possess well-established market systems and legal institutions, making them the preferred choice for new energy enterprises. Therefore, per capita GDP is selected as the measurement indicator for local economies of scale.
5. Industrial Structure: Industrial structure is a critical factor influencing firm location decisions. Given that renewable energy enterprises are significantly affected by upstream and downstream industries, the majority of their products are directed toward markets related to the tertiary sector. Consequently, the supply–demand dynamics, market opportunities, and market potential of the associated industrial chains play a decisive role in their operations. The proportion of the tertiary sector in GDP is chosen as the measurement indicator.
6. Foreign Direct Investment (FDI): Foreign direct investment serves as an indicator of a city’s level of openness to the outside world, integrating regional economies with international markets. A higher degree of openness enhances the comparative advantages of regional industries, facilitating industrial agglomeration. Renewable energy enterprises benefit from active communication and collaboration with both domestic and international partners, enabling the introduction of advanced technologies. The actual amount of foreign capital utilized in a given year is selected for this variable.
7. Total Freight Volume: In addition to HSR, transportation includes railways, highways, waterways, and civil aviation. The development and operation of these transportation infrastructures significantly influence total freight volume. Regions characterized by high freight volumes typically possess comprehensive multimodal transportation networks. These systems reduce transportation costs and significantly influence renewable energy enterprise location decisions. The total freight volume for each prefecture-level city is used as a measure.
8. Pollution Levels: As economic development progresses, there is an increasing emphasis on environmental protection and energy conservation, and people are paying more attention to the importance of low-carbon emission reduction. The development of renewable energy aligns with this perspective and positively contributes to the growth of renewable energy enterprises. Sulfur dioxide emissions are selected as the measurement for pollution levels.

In addition to the aforementioned social control variables, natural and social factors also influence enterprise location decisions. Companies must consider a region’s religion, culture, customs, climate, land availability, and local residents’ values, as well as their acceptance of the company’s products. However, since these factors are too complicate to quantify, this study employs a method to control for temporal and spatial effects. This approach aims to mitigate measurement errors arising from the omission of these natural factors or other unconsidered variables. Descriptive statistics for the variables are presented in Table 1.

**Table 1:** Descriptive statistics

Variables	N	Mean	Standard error	Min	Max
Newly established renewable energy enterprises	3525	46.4	93.7	0	1286
Opening of High-Speed Rail	3525	0.5	0.5	0	1
Population	3524	479.0	322.1	17.2	3416
Local economies of scale	3525	4.4	11.2	0	642.2
Industrial structure	3524	41.1	69.8	11.1	4139
Foreign direct investment	3525	988.3	2292	0.3	34,851
Total freight volume	3525	161.6	166.5	3.2	2111
Pollution levels	3525	52.0	56.8	0	683.2
Resource endowments	3520	16.3	30.7	0.1	1592.93

## Benchmark regression and data analysis

### Benchmark regression analysis

Based on the model, a multi-period DID analysis is conducted on the study samples. The benchmark regression results are presented in Table 2. Specifically, Column (1) shows the results without control variables, while Column (2) incorporates the aforementioned control variables. Columns (3) and (4) control for time effects and spatial effects, further reducing selection bias and time trend effects. The results demonstrate consistently positive and significant effects of HSR openings on renewable energy enterprises. This suggests that HSR promotes the growth of such enterprises, thereby validating Hypothesis 1. The subsequent analysis will delve into the mechanisms through which HSR openings influence the location choice of renewable energy enterprises.

**Table 2:** Baseline regression results

	(1)	(2)	(3)	(4)
Opening of High-speed rail	75.3912*** (4.6824)	32.8954*** (6.2585)	29.0731*** (6.3975)	11.4203*** (4.1779)
Population		0.0448*** (0.0160)	0.4094*** (0.1100)	0.4011*** (0.1058)
Local economies of scale		0.1748 (0.1905)	0.1491 (0.1412)	0.0430 (0.0964)
Industrial structure		0.0022 (0.0108)	0.0022 (0.0084)	− 0.0005 (0.0033)
Foreign direct investment		0.0167*** (0.0047)	0.0145*** (0.0047)	0.0148*** (0.0049)
Total freight volume		0.1199*** (0.0441)	0.0691 (0.0480)	0.0433 (0.0533)
Pollution levels		− 0.4193*** (0.0918)	− 0.4965*** (0.1296)	− 0.4012*** (0.1428)
_cons	9.7982*** (1.4530)	− 6.0398 (5.7443)	− 164.0161*** (51.4074)	− 158.6645*** (49.0652)
Time fixed effects	No	No	No	Yes
City fixed effects	No	No	Yes	Yes
R^2^			0.4289	0.5159
N	3525	3523	3523	3523

Standard errors in parentheses. *** represents the significance at 1% level.

### Parallel trend test

In a multi-period DID framework, the varying timing of HSR openings complicates the time trend graphs, making them challenging to interpret. Therefore, a parallel trend test specific to the multi-period DID is employed. This test examines whether there are significant differences in the location choice of renewable energy enterprises before and after the opening of HSR, thereby assessing whether the conditions for the DID model’s parallel trend assumption are met.

**Fig. 2:**
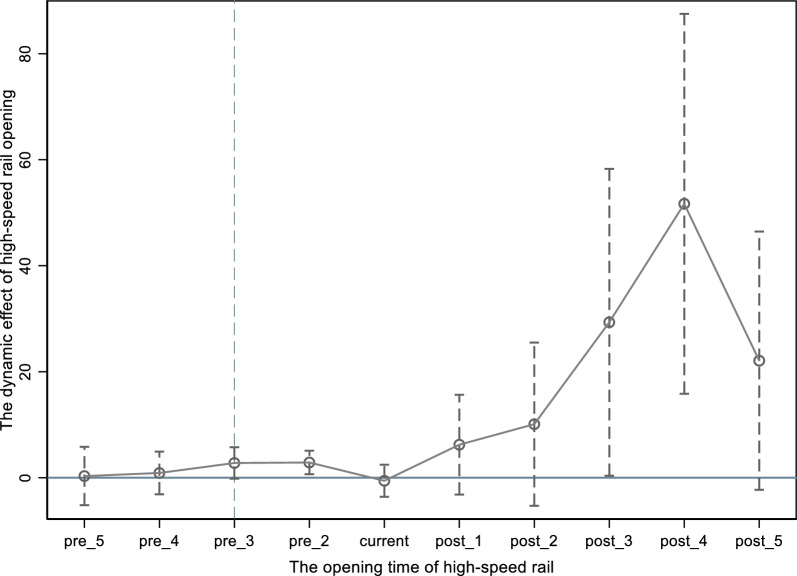
Results of parallel trend tests

According to the principles of the DID model, the development trends of the treatment group and the control group must be consistent prior to the implementation of the policy. Drawing on methods from Cai and Zhong [34], this study utilizes an event study approach to analyze the dynamic effects of HSR openings on the location choice of renewable energy enterprises. A dummy variable is constructed for the year of HSR opening, with pre_1 to pre_5 representing dummy variables relative to the year before the opening. Specifically, pre_1 indicates the year before the opening of HSR, coded as 1, while the other years are coded as 0. The remaining dummy variables follow this pattern. Similarly, post_1 to post_5 denote dummy variables for the years following the HSR opening, with the same coding principle applied. The variable “current” represents the year of the HSR opening. As illustrated in Fig. 2, the regression coefficients for all indicators before the policy implementation are not statistically significant. This indicates that there are no notable differences in site selection for renewable energy enterprises between the treatment and control groups before the opening of HSR. This finding satisfies the prerequisite for the parallel trend assumption in the multi-period DID model. After the policy is implemented, the regression coefficients begin to show positive values and a significant upward trend, further demonstrating the impact of HSR openings on the attractiveness of cities for renewable energy enterprises.

### Robustness analysis

Robustness tests evaluate the validity of the assessment methods and selected indicators. If the results of the evaluation methods and indicators remain unchanged despite variations in certain parameters, then the robustness test is passed. In examining whether the opening of HSR influences the number of renewable energy enterprises, the results will inevitably be interfered by other confounding factors. Therefore, methods such as increasing control variables and conducting counterfactual analyses are employed to test the robustness of the regression results.

1. Controlling for Resource Endowments: The various production factors available in a city—such as capital, labor, land, management, and technology—determine the types of enterprises that can be attracted to the city. Capital and labor both influence the spatial distribution of renewable energy enterprises. Thus, the ratio of labor to capital is selected to measure how differences in resource endowments affect enterprise location choices. As shown in Column (1) of Table 3, after controlling for resource endowments, the impact of HSR openings on the site selection of renewable energy enterprises remains significant.
2. Controlling for Policy Effects: Policies and resource availability are crucial factors influencing the layout of China’s renewable energy industry. With increasingly stringent environmental protection regulations in China, the government has provided various subsidies to the renewable energy sector. For example, tax incentives can reduce operational costs for enterprises, while specific investment policies offer funding support for technological research in renewable energy, thereby attracting more companies. To control for the effects of policy adjustments on the increase of renewable energy enterprises, provincial fixed effects and time effects are cross-multiplied. As shown in Column (2) of Table 3, the results remain significant even after accounting for policy effects.
3. Placebo test: To further validate the robustness of the benchmark regression results, a counterfactual analysis is conducted by altering the timing of policy implementation. The objective is to investigate whether the location choice of renewable energy enterprises is influenced by factors other than the opening of HSR. To assess the impact of these alternative factors, the opening dates of HSR in different cities are shifted earlier by 1 to 5 years, represented by the variables hasrailway_1 to hasrailway_5. In this benchmark model, new variables are incorporated for regression analysis. If the dummy variables yield significant results, it suggests that the location choice of renewable energy enterprises may be influenced by other factors. Conversely, if the results remain insignificant, it indicates that the changes in location choice are primarily attributable to the opening of HSR, thereby further validating the previous conclusions. Table 4 presents the regression results after incorporating the adjusted HSR opening dates into the model, with all regression coefficients showing no significance. This finding further verifies that the opening of HSR is a major factor influencing the location choice of renewable energy enterprises.

**Table 3:** The results of the robustness tests

	(1)	(2)
Opening of high-speed rail	11.4681*** (4.1819)	10.2099** (4.3393)
Resource endowments	0.0364* (0.0313)	0.0037* (0.0113)
Control variables	Yes	Yes
Fixed effect	Yes	Yes
Provincial-by-year fixed effects	No	Yes
R^2^	0.5164	0.7604
N	3518	3518

Standard errors in parentheses. *, **, and *** represent the significance at 10%, 5%, and 1% levels, respectively

**Table 4:** The results of placebo test

	(1)	(2)	(3)	(4)	(5)
Opening of high-speed rail_1	− 6.5064 (4.4548)				
Opening of high-speed rail_2		− 16.4095 (4.5512)			
Opening of high-speed rail_3			− 20.4629 (4.4995)		
Opening of high-speed rail_4				− 21.9119 (4.3288)	
Opening of high-speed rail_5					− 21.7993 (4.1999)
Control variables	Yes	Yes	Yes	Yes	Yes
Fixed effect	Yes	Yes	Yes	Yes	Yes
Provincial-by-year fixed effects	Yes	Yes	Yes	Yes	Yes
R^2^	0.7597	0.7622	0.7637	0.7640	0.7636
N	3518	3518	3518	3518	3518

### Heterogeneity analysis

Heterogeneity analysis assesses the extent of variation in effect sizes across a series of studies, highlighting the presence of differences beyond predictable factors. Different enterprises operate under varying conditions, which can lead to differing impacts on regression results. Heterogeneity analysis will be used to further study the results.

1. Geographical Heterogeneity Analysis: Differences in regional geographical locations may result in varying effects of HSR on the location choice of renewable energy enterprises. Cities across China are categorized into three regions: Eastern, Central, and Western. This allows for an examination of the differential impact of HSR openings on the number of renewable energy enterprises across these regions. Here, hasrailway_east represents the interaction term between the Eastern region and the opening of HSR. Similarly, hasrailway_mid and hasrailway_west are the interaction terms for the Central and Western regions, respectively.^6^ The moderating effect of geographical differences on enterprise location selection can be observed through regression results. The findings presented in Table 5 indicate that HSR openings have a significant positive effect on cities in the Eastern region. In contrast, the impact is less pronounced for cities in the Central and Western regions. This difference may be attributed to the lower levels of economic and technological development in the Central and Western regions, which are not attractive to renewable energy enterprises. Additionally, these regions may not have adequately leveraged their natural resource advantages.
2. Heterogeneity Analysis of Urban Development Levels: There exists a phenomenon of unbalanced development among different cities. The size and economic status of a city may moderate the relationship between the opening of HSR and the number of renewable energy enterprises. To assess urban development levels, cities are categorized based on their Gross Domestic Product (GDP): those in the top third are regarded as well-developed cities, those in the middle third as moderately-developed cities, and those in the bottom third as less-developed cities. The interaction terms between HSR openings and these dummy variables are represented by hasrailway_high, hasrailway_med, and hasrailway_low, respectively. The heterogeneity analysis presented in Table 5 indicates that well-developed cities exhibit a greater receptivity to renewable energy enterprises, while moderately- and less-developed cities show a certain crowding-out effect. Notably, the crowding-out effect is more pronounced in less-developed cities compared with moderately-developed ones. This suggests that renewable energy enterprises tend to favor cities with higher levels of economic development. The heterogeneity analysis based on urban development levels thus validates Hypothesis 2.
3. Heterogeneity Analysis of Environmental Awareness: To establish a green, low-carbon circular economy, urban environmental awareness is critically important, as the settlement of renewable energy enterprises is closely linked to this awareness. This analysis evaluates three indicators of environmental consciousness: the comprehensive utilization rate of industrial solid waste, the urban sewage treatment rate, and the harmless treatment rate of domestic garbage. Cities with above-average levels on these indicators are classified as having strong environmental awareness and coded as 1, whereas those below average are coded as 0. The interaction terms between HSR openings and these three dummy variables are represented by hasrailway_solid, hasrailway_sewage, and hasrailway_garbage, respectively. The results from the heterogeneity analysis of environmental awareness, presented in Table 5, reveal a positive impact of HSR openings on the number of renewable energy enterprises. This indicates that cities with higher environmental awareness attract more renewable energy companies. Hence, the heterogeneity analysis regarding environmental awareness confirms Hypothesis 3.

**Table 5:** Test results of heterogeneity analysis

Variables	(1)	Variables	(2)	Variables	(3)
Opening of high-speed rail_east	55.05*** (11.48)	Opening of high-speed rail_high	56.08*** (11.61)	Opening of High-speed rail_solid	24.10*** (8.112)
Opening of high-speed rail_mid	15.86** (8.042)	Opening of high-speed rail_med	7.943 (6.888)	Opening of high-speed rail_sewage	44.65*** (8.060)
Opening of high-speed rail	45.44*** (6.984)	Opening of high-speed rail	52.88*** (4.360)	Opening of high-speed rail	39.55*** (4.792)
Constant	8.746*** (2.281)	Constant	8.606*** (2.320)	Constant	8.857*** (2.232)
Control variables	Yes		Yes		Yes
Fixed effect	Yes		Yes		Yes
Provincial-by-year fixed effects	Yes		Yes		Yes
Observations	3525	Observations	3525	Observations	3525
R-squared	0.225	R-squared	0.229	R-squared	0.232

Standard errors in parentheses. ** and *** represent the significance at 5% and 1% levels, respectively.

### Endogeneity analysis

Given that urban R&D expenditure may potentially exert influence on renewable energy enterprise location decisions, we implemented an endogeneity test utilizing this variable. As presented in Table 6, the coefficient for HSR inauguration maintains statistical significance after incorporating urban R&D expenditure controls. This demonstrates that HSR operations retain a significant impact on new energy enterprise site selection, affirming the robustness of our core findings.

**Table 6:** Test results of endogeneity analysis

Variables	(1)	(2)	(3)	(4)
Opening of high-speed rail	72.363*** (15.70)	34.866*** (6.19)	29.211*** (4.39)	11.422*** (2.81)
R&D expenditure	0.000*** (2.99)	0.000** (2.32)	0.000 (1.43)	0.000 (1.31)
Population		0.023* (1.93)	0.403*** (2.99)	0.414*** (3.11)
Local economies of scale		0.134 (0.84)	0.133 (1.02)	0.041 (0.45)
Industrial structure		− 0.000 (− 0.02)	0.002 (0.19)	− 0.000 (− 0.12)
Foreign direct investment		0.013*** (3.66)	0.014*** (3.56)	0.015*** (3.62)
Total freight volume		0.151*** (3.42)	0.107** (1.97)	0.052 (0.93)
Pollution levels		− 0.451*** (− 5.13)	− 0.499*** (− 4.20)	− 0.365*** (− 2.97)
_cons	− 7.382* (− 1.71)	− 3.544 (− 0.79)	− 174.926*** (− 2.79)	− 176.761*** (− 2.88)
Time fixed effects	No	No	No	Yes
City fixed effects	No	No	Yes	Yes
R^2^			0.478	0.548
N	3288	3288	3288	3288

Standard errors in parentheses. *, **, and *** represent the significance at 10%, 5%, and 1% levels, respectively

## Conclusion and suggestions

### Research conclusions

This study analyzes a sample of 235 cities from 2006 to 2020 utilizing the opening of high-speed rail (HSR) as a dummy variable. The analysis incorporates a comprehensive set of control variables: population, regional economic scale, industrial structure, foreign direct investment, total freight volume, and pollution levels. Through parallel trend tests, robustness analyses, and heterogeneity assessments, the study investigates the impact of HSR openings on the location choice of renewable energy enterprises. The findings are summarized as follows:

Firstly, the coefficient for the impact of HSR openings on the site selection of renewable energy enterprises is significantly positive, indicating that the HSR services have notably enhanced cities’ receptivity to renewable energy enterprises.

Secondly, heterogeneity analysis reveals that the positive influence of HSR on renewable energy enterprises varies by region and geography. Economically developed cities demonstrate a greater acceptance of renewable energy enterprises compared with less developed cities. Additionally, the number of renewable energy enterprises established in the Eastern region exceeds that in the Central and Western regions. Cities with higher waste treatment rates exhibit stronger environmental awareness, leading to a greater number of renewable energy enterprises in these areas. Overall, the analysis indicates that the development of renewable energy enterprises across China is characterized by significant imbalances.

### Policy suggestions

The positive role of transportation infrastructure, such as HSR, in economic development and industrial agglomeration is widely recognized. By utilizing the influence of HSR on the site selection of renewable energy enterprises, a more balanced development can be achieved in this sector, thereby driving regional economic growth.

First, it is essential to strategically plan HSR lines to stimulate economic development in surrounding areas. The HSR lines connects multiple cities, facilitating the movement of people and goods between them, which in turn promotes economic growth. The planning of HSR routes should aim to connect economically developed areas with underdeveloped ones, allowing advanced technologies from developed regions to flow into less developed regions where they can be applied in production processes. This connectivity can help spur economic growth in underdeveloped areas. Additionally, the HSR enables the mobility of labor, facilitating the movement of talent from developed areas to underdeveloped regions, thereby assisting these areas in addressing their developmental challenges.

Second, it is necessary to leverage regional advantages to promote the establishment of renewable energy enterprises. Well-planned HSR routes enhance the flow of labor and resources for these enterprises. Regions with sound HSR networks tend to be more economically developed and host a greater number of renewable energy companies. In contrast, areas with limited HSR connectivity often experience insufficient economic development and see fewer renewable energy enterprises. To achieve balanced development in the renewable energy sector, developed areas can transport resources and skilled personnel to underdeveloped regions through HSR to promote the economic development of these regions. Technical professionals can fully exploit the geographical advantages of less-developed areas, such as abundant resources and ample sunlight, to establish renewable energy enterprises tailored to the specific conditions of those regions.

Third, it is important to leverage the transportation advantages of HSR to promote balanced development among renewable energy enterprises. These enterprises typically prefer economically developed regions, which often have more advanced waste treatment technologies. However, such regions may also face challenges related to limited resources and constrained development space. In contrast, less-developed regions usually possess abundant natural resources, which can provide ample space for the growth of renewable energy enterprises. The advanced technologies and human resources for renewable energy development in developed regions can be transferred to underdeveloped regions through HSR, thus fully exploiting the natural resources available there and facilitating the growth of renewable energy enterprises. Furthermore, waste generated during the production processes of these enterprises can be transported via HSR to regions with more advanced waste treatment technologies for proper processing. This not only fosters balanced development across regions but also contributes to the economic growth of underdeveloped areas. Additionally, it allows for the effective utilization of locational advantages across different regions, enhancing overall development efficiency.

This study focuses on the impact of HSR openings on the site selection of renewable energy enterprises and fails to consider the potential effects on carbon emissions in the selected regions. To address this deficiency, in future studies, we will refer to the practices of Tanaka et al. [20] and Zhao et al. [28], and adopt the Multi-regional Input–output Model and the CGE Model to analyze the carbon emission issue.

## Data Availability

All relevant data supporting the findings of this study are available on request.

## Footnotes

1 Data Source: The Central People’s Government of the People’s Republic of China. https://www.gov.cn/ztzl/2008-01/25/content_868572.htm.

2 Data source: TGV official website. https://www.tgv-lyria.com/fr/en.

3 Data source: ICE official website. https://www.interrail.eu/en.

4 Data source: The Central People's Government of the People’s Republic of China. https://www.gov.cn/.

5 Data source: The Central People's Government of the People’s Republic of China. https://www.gov.cn/.

6 Specifically, hasrailway_east includes cities located in Beijing, Fujian, Guangdong, Hainan, Hebei, Jiangsu, Liaoning, Shandong, Shanghai, Tianjin, Zhejiang. hasrailway_mid includes cities located in Anhui, Henan, Heilongjiang, Hubei, Hunan, Jilin, Jiangxi, Inner Mongolia and Shanxi. hasrailway_west includes cities located in Gansu, Guangxi, Guizhou, Ningxia, Qinghai, Shaanxi, Sichuan, Xinjiang and Yunnan.
